# The role of the COVID-19 impersonal threat strengthening the associations of right-wing attitudes, nationalism and anti-immigrant sentiments

**DOI:** 10.1007/s12144-023-04305-w

**Published:** 2023-02-02

**Authors:** Anna Panzeri, Giuseppe Mignemi, Giovanni Bruno, Umberto Granziol, Cecilia Scalavicci, Marco Bertamini, Kate Mary Bennett, Andrea Spoto, Giulio Vidotto

**Affiliations:** 1 Department of General Psychology, University of Padova, via Venezia 8, Padova, Italy; 2 Department of Psychology, University of Liverpool, L69 3BX Liverpool, UK

**Keywords:** Existential threat, Anxiety, Right-Wing authoritarianism, Social Dominance Orientation, Health psychology

## Abstract

Literature showed that the link between right-wing attitudes and ethnocentric attitudes gets stronger under existential threats, but the role exerted by an impersonal threat – as COVID-19 – on right-wing attitudes is still unclear. This study aimed to highlight the role of anxiety exerted by the impersonal COVID-19 threat on the relationship between right-wing attitudes and ethnocentric attitudes, as nationalism and anti-immigrants’ sentiments. As part of an international project to evaluate the impact of COVID-19, this study administered an online survey to a representative sample (n 1038). The anxiety generated by an impersonal threat as COVID-19 – thus not exerted by any outgroup – can moderate the relationship among personal Right-Wing Authoritarianism, social dominance orientation, and ethnocentric attitudes. This is the first study demonstrating that existential threat is effective also when exerted by an impersonal agent (as COVID-19) rather than by an outgroup. Second, these findings disclose useful implications for preventive psychological interventions and for social policy makers.

**Supplementary information:**

The online version contains supplementary material available at 10.1007/s12144-023-04305-w.

## Introduction

Several studies in the scientific literature highlighted the link between the perception of an existential threat and conservative attitudes such as Right-Wing Authoritarianism (RWA) and Social Dominance Orientation (SDO; i.e., “*conservative shift*”) (Sibley & Duckitt, 2008; Duckitt, 2013; Jost et al., 2017; Perry et al., 2013).

Referring to the Duckitt’s dual-process motivational model (Duckitt, 2001, 2009) as an overarching theory, certain personality traits and the exposure to certain environmental contexts can affect a person’s view of the world and strengthen RWA and SDO.

According to Kossowska, RWA is an *“ideological response intended to reduce high levels of perceived threat and anxiety*” (2011, p. 247). RWA is an ideological belief system (Jedinger & Eisentraut, 2020) characterized by uncritical and unconditional submission to the authorities of one’s group, aggression towards individuals or groups that violate the values ​​and norms of the in-group, as well as by conventionalism and unconditional adherence to values ​​and norms of own group (Altemeyer, 1981; Kossowska et al., 2011).

Differently, SDO goes beyond, it is characterized by a strong opposition to equality between social groups (i.e., anti-egalitarianism) and support for dominance by high-status groups towards low-status groups (Dominance; Ho et al., 2015).

Moreover, RWA and SDO, rooted in two different worldviews, predict different attitudes and behaviors towards the outgroup.

The perception and belief of the world as a dangerous threat leads to RWA, and high levels of RWA predict high levels of prejudice towards dangerous social groups that threaten the health and survival of the in-group. Noteworthy, the level of RWA predicts less favorable attitudes towards the out-group only when this is perceived as an economic or cultural threat (Duckitt & Sibley, 2010).

Similarly, the view of the world as a competitive jungle leads to SDO (Perry et al., 2013; Sibley & Duckitt, 2008), and high levels of SDO are associated with negative attitudes towards out-groups that undermine the hierarchical order of social groups. Importantly, SDO predicts negative attitudes regardless of the perception of threat (Duckitt & Sibley, 2010).

The levels of RWA and SDO are considered rather stable over time, but they can vary in relation to certain situational factors: the perception of threat influences RWA, and competition between groups influences SDO (Duckitt, 2001, 2009; Duckitt et al., 2010; Duckitt & Sibley, 2009).

Scientific literature highlighted a complex relation among perceived threats and authoritarianism, ethnocentrism, and prejudice (Adorno et al., 2019; Altemeyer, 1998; Bizumic & Duckitt, 2012; Duckitt, 2020; Osborne et al., 2017). Importantly, different types of threats may influence authoritarianism (Butler, 2013) and influence RWA levels (Duckitt & Fisher, 2003; Sales & Friend, 1973).

Regarding the characteristics of threat, the threats towards the in-group are those able to strongly predict higher levels of authoritarianism (Feldman, 2013; Shaffer & Duckitt, 2013).

Moreover, the existential threat has a greater impact on right-wing attitudes rather than threats related to economic resources (Merolla & Zechmeister, 2009). According to some studies, a perceived existential threat strengthens (i.e., *moderates*) the association between RWA and prejudicial attitudes towards the out-group (Caricati et al., 2017; Echebarria-Echabe & Fernández-Guede, 2006; Mancini et al., 2020; Mirisola et al., 2014).

Interestingly, the threat versus the in-group can be both realistic (i.e., a threat to physical health, material and/or economic resources) or symbolic (i.e., a threat to moral beliefs or in-group values) (Jedinger & Eisentraut, 2020; Stephan et al., 2009).

In recent years, in Italy several studies have been conducted on the role of RWA on inter-group attitudes (Boin et al., 2020; Caricati et al., 2017; Chirumbolo et al., 2016; Manganelli Rattazzi et al., 2007; Roccato et al., 2021), showing how people with high levels of RWA are more likely to believe that immigrants represent a threat to their in-group (Mancini et al., 2020) and have less positive attitudes towards out-group members (Mirisola et al., 2014; Passini, 2017).

Recently, the pandemic had a considerable negative impact for individual and public health, as well as on the stability of the economic system and social order (Cerami et al., 2020; Mignemi et al., 2022; Panzeri & Ferrario, 2020; Panzeri et al., 2021c; Roccato et al., 2021; Rossi et al., 2021; Rossi Ferrario et al., 2021).

Considering the abovementioned reasons, the unexpected COVID-19 pandemic represents a very serious and persistent existential threat, with an important impact on intergroup dynamics, attitudes, and behaviors.

Notably, the pandemic is a generic impersonal threat not exerted by any out-group (Hartman et al., 2021; Kossowska et al., 2011), it represents an unprecedented scenario to study the psychological effects of an impersonal existential threat. Indeed, to date, literature has highlighted the association between the existential threat exerted by a group, political ideology (i.e., RWA and SDO), and intergroup attitudes (i.e., ingroup favoritism and outgroup derogation; Duckitt, 2020; Feldman, 2003), but little is known about a generic impersonal threat not referable to any specific social group (i.e., COVID-19).

To this aim, the abovementioned Duckitt’s dual process model is considered as an overarching framework both for variable selection and for the interpretation of the results.

Few studies from different countries (e.g., UK, Ireland, Canada, and Japan) suggested how the current pandemic may have a negative effect on attitudes toward immigrants (Esses & Hamilton, 2021; Hartman et al., 2021; Newbold et al., 2021; Yamagata et al., 2020). However, to date, no studies have been carried out in Italy on the role of RWA and the pandemic threat in predicting attitudes toward immigrants. Literature still lacks clear evidence explaining how the perception of the health threat linked to COVID-19 plays a role in determining high levels of prejudice towards the out-group in Italy.

Given this background, the present study explores for the first time the role of an existential threat caused by the pandemic in moderating the relationship between individual-level right-wing (RWA and SDO) and prejudicial attitudes towards immigrants in the Italian context.

This research question is important because some contextual factors, independently from socio-psychological trait predispositions, may lead people to systematically shift toward right-wing attitudes. Importantly, understanding these phenomenological dynamics may represent a starting point to inform and realize interventions aimed to favor inclusive attitudes towards the outgroup to improve the social good (Bochicchio et al., 2021).

The variables were chosen in view of the Duckitt’s dual process model as a theoretical framework, namely, RWA and SDO were considered as independent variables, and nationalism and anti-immigrant sentiments were the dependent variables. Also, COVID-19 anxiety was chosen to represent the existential threat.

It is possible to hypothesize that high levels of a perceived impersonal threat (not attributable to a social group) as COVID-19 may interact with RWA in predicting stronger ethnocentric attitudes (i.e., nationalism and anti-immigrant sentiments) in the Italian general population.

## Methods and materials

This study is part of the international ‘COVID-19 Psychological Research Consortium’ (C19PRC), a project launched in March 2020 in the United Kingdom (UK) (McBride et al., 2020a) to understand the psychosocial impact of COVID-19 across several countries (Shevlin et al., 2022). As part of an international consortium sharing the same aims, the survey of the Italian branch (https://osf.io/qy65b/) of the Consortium was in line with the surveys from other countries (McBride et al., 2020b).

### Study plan

The present study relied on an online survey administered in four Italian regions – Campania, Lazio, Lombardia, and Veneto. The inclusion criteria were living in one of the 4 selected regions and being at least 18 years old. A stratified quota sampling was used to guarantee that the sample was representative of the Italian population (gender, age, household income, and region). All participants provided informed consent before completing the survey. Ethical approval was provided by the Ethical Committee for Psychological Research of the University of *[blinded for review]* (protocol number 3818).

Data were collected from July 13th to July 28th, 2020, after the contagion peak (end of March) and after the end of the strict national lockdown (May 18th ). At that time, in Italy, a total of 243,230 cases of COVID-19 had been registered, with almost 35,000 deaths. Lombardia and Veneto, in the north of the country, were the regions with the highest number of contagions while in the center (e.g., Lazio) and south (e.g., Campania) the outbreak was more contained.

### Participants

Data from 1038 adult participants were recruited for this study via Qualtrics from an online research panel. The median time of completion of the survey was 41 min.

The mean age of the total sample was 49.94 years (median = 51, SD = 16.14, range = 18–87), and 51.15% were female (*n* = 531). Participants were enrolled from the 4 selected regions based on their population size: Campania (*n* = 227), Lazio (*n* = 234), Lombardia (*n* = 391), Veneto (*n* = 186). Most of them were Italian (96.61%, *n* = 1003) and with Caucasian ethnicity (74.66%, n = 775). Nearly half of the sample had completed at least high school (48.74%, *n* = 506) with a further 42.97% having a higher level of education. Less than half were fully employed (44.41%, *n* = 461), with 24.18% retired (*n* = 251).

Only 14 participants reported that they had tested positive for COVID-19 (1.35%), and 10.50% said they were mourning a loss due to confirmed cases of COVID-19. Further information about demographics and variables is available in the supplementary materials (SI, Tables S1-S20).

### Materials

The measures relevant to the present analysis are listed and described below. The full list is available as supplementary material. The whole survey was in Italian.

*Sociodemographic variables*: we collected the same information as the original C19PRC-UK study: gender, age, educational level, income, and previous health issues. We integrated these with further information (region, losses due to COVID-19, perceived risk to contract COVID-19).

*Educational* level was rescaled and dummy coded in low/moderate levels through the median-split procedure. Gross household *income* was kept continuous and rescaled from 0 to 1. The exact wording for these and other psychological measures and the complete Italian survey (Bruno et al., 2020), as well as descriptive statistics, are available in the Supplemental Appendix.

*Nationalism*: in line with the original study, Italian nationalism was assessed by two items adapted from Davidov (2011) on a 5-point Likert scale: (1) *“The world would be a better place if people from other countries were more like the Italian”* and (2) *“Generally speaking, Italy is a better country than most other countries”*. The items were combined and rescaled from 0 to 1 (1 = higher nationalism). The standardized Cronbach’s alpha was 0.76.

*Anti-immigrant sentiment*: two items from the British Social Attitudes Survey (2015) were translated to assess attitudes toward migrants on a 10-point scale: (1) “Would you say it is generally bad or good for Italian’s economy that migrants come to Italy from other countries?” and (2) “Would you say that Italian’s cultural life is generally undermined or enriched by migrants coming to live here from other countries?”. The two items were reverse coded and rescaled from 0 to 1 (1 = higher anti-immigrant sentiment). The standardized Cronbach’s alpha was 0.88.

*RWA*: The six-item Very Short Authoritarianism Scale (Bizumic & Duckitt, 2018) was used to assess participants’ levels of RWA. Responses were collected on a 5-point Likert scale. These six items were combined and rescaled from 0 to 1 (1 = higher authoritarianism). The reliability was satisfactory with a value of 0.67.

*SDO*: The 8-item Social Dominance Orientation scale (Aiello et al., 2019; Ho et al., 2015) was used to assess participants’ levels of social dominance. Responses were collected on a 5-point Likert scale. These eight items were combined and rescaled from 0 to 1 (1 = higher social dominance). The Cronbach’s alpha was good with 0.75.

*Political and ideological orientation*: As in the original study, three 10-point scale questions were adapted from the 2014 to 2023 British Election Study (2017). The focus was on the self-description of (i) participants’ political orientation (10 = right-wing), (ii) ideological orientation toward fiscal issues (e.g., taxes; 10 = very conservative) and (iii) ideological orientation toward social issues (e.g., abortion; 10 = very conservative). Each question was rescaled ranging from 0 to 1. The standardized Cronbach’s Alpha was 0.78.

*Conspiracy beliefs related to COVID-19*: 5 visual analog scales (VAS) from 0 (= ‘I do not believe it at all’) to 100 (= ‘I believe it totally’) were used to measure the adherence of each participant to conspiracy beliefs related to COVID-19. An example of an item was *‘The 5G networks are the real responsible for the current pandemic’*. The standardized Cronbach’s alpha was acceptable with a value equal to 0.71.

*COVID-19-related anxiety*: was measured as a single item, ‘How anxious are you about the coronavirus COVID-19 pandemic?’ and rated on a continuous scale from 0 to 100. The item was rescaled from 0 to 1 (1 = higher COVID-19 related anxiety).

### Statistical analyses

First, the relationships among demographics, socio-political variables, and psychological constructs were explored through Spearman’s correlation coefficients, the associated *p*-values were adjusted controlling for the false discovery rate (Benjamini & Hochberg, 1995).

Second, the ordinary least squares linear regression was used to test the role of perceived COVID-19 anxiety threat (as a moderator) on the association between authoritarianism (i.e., RWA, independent variable) and political attitudes (dependent variables). So, three regression models were fitted, each with a different dependent variable: nationalism; anti-immigrant sentiments about the Economy; and anti-immigrant sentiments about Culture. The predictor variables were always RWA, SDO, COVID-19 anxiety, the interactions of COVID-19 anxiety with RWA and SDO. We will refer to these as the ‘unadjusted’ models as we did not include socio-political covariates. All variables used in the regression models were standardized through a rescaling from 0 to 1.

After, to control for the effect of the sociodemographic and political orientation covariates (e.g., age, gender, region, conservativism, conspiracy, political views), we estimated the same models by adding such covariates, we will call these the ‘adjusted’ models.

To test the region of the significance of the interaction, we used the Johnson-Neyman interval analysis (Johnson & Neyman, 1936) to test for which values of the moderator the relationship between predictors and dependent variables was statistically significant.

The alpha level was set at 0.05, only results with an associated *p*-value below this threshold are commented as statistically significant – also other results not statistically significant results are described. All the *p*-values were adjusted for the false discovery rate (Benjamini & Hochberg, 1995), representing the expected proportion of false discoveries among the rejected hypotheses. The R Core Team Software was used for all the statistical analyses (R Core Team, 2018). The *ggplot2* package was used for the graphs (Wickham, 2016).

## Results

Table 1 shows the Spearman’s *r* correlations among all variables, the *p*-values are adjusted to control for the false discovery rate (Benjamini & Hochberg, 1995). Right-wing political orientation, social and fiscal conservatism, authoritarianism, and social dominance were positively and significantly correlated with nationalism, as well as with anti-immigrant sentiment related to the internal economy and national culture. Interestingly, COVID-19 conspiracy beliefs showed a positive and significant correlation with conservative orientation and right-wing ideology. We can see that COVID-19 anxiety has very small and not statistically significant associations with Nationalism (r = .02, *p* = .582), Economy (r = − .05, *p* = .200), Culture (r = − .05, *p* = .135), RWA (r = .03, 0.476), and SDO (r = .02, *p* = .667).

**Table 1:**
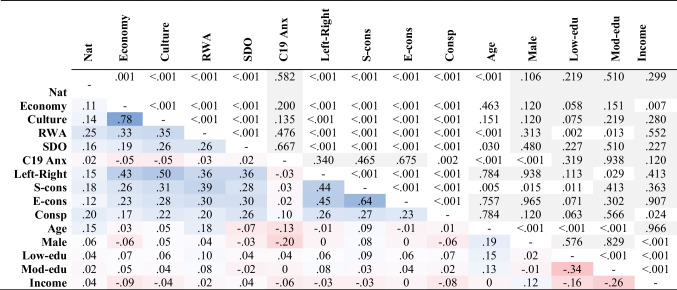
Spearman’s correlations

The lower triangle contains the correlation index. The blue color indicates positive correlations and the red color indicates negative correlations. More intense colors indicate a stronger correlation. The upper triangle contains the p-value adjusted for the Benjamini-Hochberg method. The light-grey cells contain the non-significant *p*-values.

Nat = Nationalism; RWA = Right-wing authoritarianism; SDO = Social dominance orientation; C19Anx = COVID-19 anxiety; Left-Right = Left-Right political view; S/E-cons = Social/Economic conservatism; Cons*p* = Conspiracy; Low/Mod-edu = Low/Moderate education

Then, ordinary least squares regression was chosen to test the role of perceived COVID-19 anxiety threat on the association between authoritarianism and political attitudes. All variables in the regression models were standardized with a rescaling from 0 to 1. Nationalism and anti-immigrant sentiments (on Economy and Culture) were separately regressed on RWA, SDO, COVID-19 anxiety – and its interaction with RWA and SDO – and the Italian regions.

Table 2 shows the results of regression models, reporting both ‘unadjusted’ and ‘adjusted’ estimates – after the introduction of the socio-demographic and political orientation as covariates.

**Table 2:** Results of the ordinary least squares regressions models

			Dependent variables			
	Nationalism		Economy		Culture	
	Estimates		Estimates		Estimates	
Predictors	Unadj,	Adj.	Unadj,	Adj.	Unadj,	Adj.
RWA	0.28* [0.11, 0.46]	0.18 [0.00, 0.36]	0.24 [0.00, 0.48]	0.05 [-0.18, 0.28]	0.40* [0.17, 0.64]	0.16 [-0.06, 0.39]
	*p* < .01	*p* = .15	*p* = .05	*p* = .89	*p* < .01	*p* = .29
SDO	− 0.13 [-0.31, 0.06]	− 0.10 [-0.29, 0.08]	0.35* [0.10, 0.60]	0.25 [0.01, 0.49]	0.30* [0.05, 0.55]	0.18 [-0.06, 0.41]
	*p* = .23	*p* = .57	*p* = .01	*p* = .09	*p* = .03	*p* = .29
C19 anxiety	− 0.19* [-0.35, − 0.03]	− 0.16* [-0.31, 0.00]	− 0.32* [-0.54, − 0.11]	− 0.27* [-0.47, − 0.06]	− 0.31* [-0.53, − 0.10]	− 0.20* [-0.40, − 0.01]
	*p* = .03	*p* < .001	*p* = .01	*p* = .04	*p* = .01	*p* = .25
RWA: C19 anxiety	0.10 [-0.20, 0.39]	0.13 [-0.16, 0.42]	0.68* [0.29, 1.07]	0.65* [0.28, 1.02]	0.40 [0.01, 0.79]	0.36 [-0.01, 0.72]
	*p* = .52	*p* = .66	*p* < .002	*p* < .003	*p* = .06	*p* = .25
SDO: C19 anxiety	0.47* [0.17, 0.78]	0.35 [0.05, 0.65]	− 0.26 [-0.67, 0.14]	− 0.40 [-0.79, − 0.02]	0.08 [-0.32, 0.50]	− 0.09 [-0.48, 0.29]
	*p* < .01	*p* = .11	*p* = .21	*p* = .09	*p* = .66	*p* = .76
Region Lazio	-	0.01 [-0.03, 0.05]	-	0.04 [-0.01, 0.09]	-	0.03 [-0.02, 0.08]
		*p* = .96		*p* = .29		*p* = .31
Region Lombardia	-	0.00 [-0.03, 0.04]	-	0.03 [-0.02, 0.07]	-	0.04 [-0.00, 0.09]
		*p* = .98		*p* = .46		*p* = .25
Region Veneto	-	0.00 [-0.04, 0.04]	-	0.01 [-0.05, 0.06]	-	− 0.01 [-0.07, 0.04]
		*p* = .98		*p* = .89		*p* = .76
Societal Conservatorism	//	0.00 [-0.01, 0.01] *p* = .98	//	0.01 [-0.00, 0.01] *p* = .31	//	0.01 [-0.00, 0.01] *p* = .29
Economic Conservatorism	//	0.00 [-0.01, 0.01] *p* = .97	//	− 0.00 [-0.01, 0.01] *p* = .62	//	− 0.00 [-0.00, 0.01] *p* = .76
Conspiracy	//	0.19* [0.12, 0.26] *p* < .001	//	− 0.00 [-0.09, 0.09] *p* = .99	//	0.05 [-0.04, 0.14] *p* = .39
Political views (1 = right, 0 = left)	//	0.00 [-0.00, 0.01] *p* = .96	//	0.04* [0.03, 0.05] *p* < .001	//	0.05* [0.04, 0.05] *p* < .001
Age	//	0.11* [0.05, 0.17] *p* = .001	//	− 0.01 [-0.09, 0.07] *p* = .89	//	0.01 [-0.06, 0.09] *p* = .83
Male	//	0.02 [-0.01, 0.04] *p* = .46	//	− 0.04 [-0.08, − 0.01] *p* = .07	//	0.03 [-0.01, 0.06] *p* = .29
Income	//	0.03 [-0.01, 0.07] *p* = .46		− 0.08* [-0.13, − 0.03] *p* = .02	//	− 0.05 [-0.10, 0.00] *p* = .25
Low Educational Level	//	− 0.00 [-0.05, 0.05] *p* = .98	//	0.01 [-0.06, − 0.07] *p* = .89	//	− 0.01 [-0.07, 0.06] *p* = .88
Mod Educational Level	//	0.00 [-0.03, 0.03] *p* = .98		− 0.01 [-0.04, -03] *p* = .89	//	− 0.01 [-0.04, 0.03] 0.83
F	21.26*	9.50*	34.41*	20.06	42.52*	26.45*
Adjusted R ^2^	0.09	0.12	0.14	0.24	0.17	0.30

* = *p* < .05 (all *p* values are adjusted controlling the false discovery rate)

The Unadj. columns refer to the models without the socio-political covariates, the Adj. models refer to the models adjusted including the socio-political covariates

In each cell (i) the unstandardized estimates, (ii) the 95% confidence intervals in brackets, and (iii) the rounded p-value are reported

RWA: Right-wing authoritarianism; SDO: Social dominance orientation

As reported in Table 2, the main regressions’ results are the following. RWA has a significant effect on nationalism (unadj: B = 0.28, *p* < .01), and anti-immigrant sentiments about culture (unadj: B = 0.40, *p* < .01). SDO has a significant effect on the anti-immigrant sentiments about economy (unadj: B = 0.35, *p* = .01) and the anti-immigrant sentiments about culture (unadj: B = 0.30, *p* = .03). COVID-19 anxiety has a significant main effect on nationalism (unadj: b = − 0.19, *p* = .03; adj: B = − 0.16, *p* < .001), anti-immigrant sentiments about economy (unadj: B = − 0.32, *p* = .01; adj: B = − 0.27, *p* = .04), and anti-immigrant sentiments about culture (unadj: B = − 0.31, *p* = .01).

About the interactions, the interaction of COVID-19 anxiety and RWA has a significant effect on anti-immigrant sentiments about the economy both in the model without covariates (unadj: B = 0.68, *p* = 001) and with covariates (adj: B = 0.65, *p* < .003) – and its effect on anti-immigrant sentiments about culture is positive but not significant (unadj: B = 0.40, *p* = .055; adj: B = 0.36, *p* = .25). The interaction of COVID-19 anxiety and SDO has a significant effect on nationalism (unadj: B = 0.47, *p* < .01).

About the socio-political covariates in the adjusted models, both societal and economic conservativism do not have a significant effect on the dependent variables. Nor the geographical region doesn’t have any significant effect. Conspiracy has a significant effect on nationalism (adj: B = 0.19, *p* < .001). The (right) political views have a small positive effect on anti-immigrant sentiments about the economy (adj: B = 0.04, *p* < .001) and culture (adj: B = 0.05, *p* < .001). Age has a significant positive effect on nationalism (adj: B = 0.11, *p* = .001). The income has a significant negative effect on anti-immigrant sentiments about the economy (adj: B = − 0.08, *p* = .02). The educational level does not have an effect on the dependent variables.

**Fig. 1:**
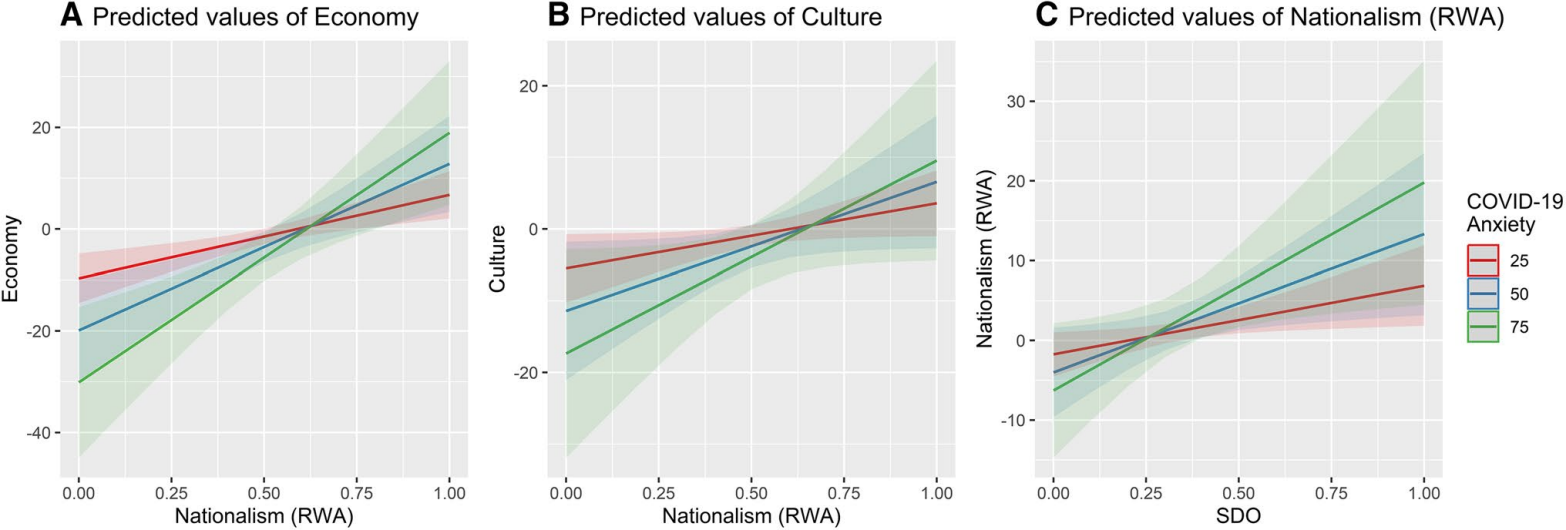
Interactions in the regression models with covariates. On the x-axis the independent variable that influences the dependent variable on the y-axis, such effect is moderated by the levels of COVID-19 anxiety. Note: the conditional effect of right-wing authoritarianism (RWA) on anti-immigrant sentiments related to the internal economy (**A**) and national culture (**B**) at different levels of COVID-19 anxiety. **C** the conditional effect of social dominance orientation (SDO) on nationalism at different levels of COVID-19 anxiety. Each plot shows the estimated conditional effect on each outcome at different levels of anxiety on the adjusted estimates (models with covariates)

Relying on these regressions, Fig. 1 shows some interactions of COVID-19 anxiety with RWA and SDO in the models adjusted with covariates. It is possible to observe that the COVID-19 anxiety moderates the relationships between RWA and anti-immigrant sentiments about the economy, RWA and anti-immigrant sentiments about culture, and SDO and nationalism. In other words, at different levels of the moderator, the independent variables have a different effect on the dependent variables.

To deepen the moderation effect of COVID-19 anxiety in the models with covariates, the Johnson-Neyman interval was calculated to test at which values of the moderator – COVID-19 anxiety, with observed values ranging from 0 to 1 – the slope of the predictor on the predicted variable is statistically significant.

**Fig. 2:**
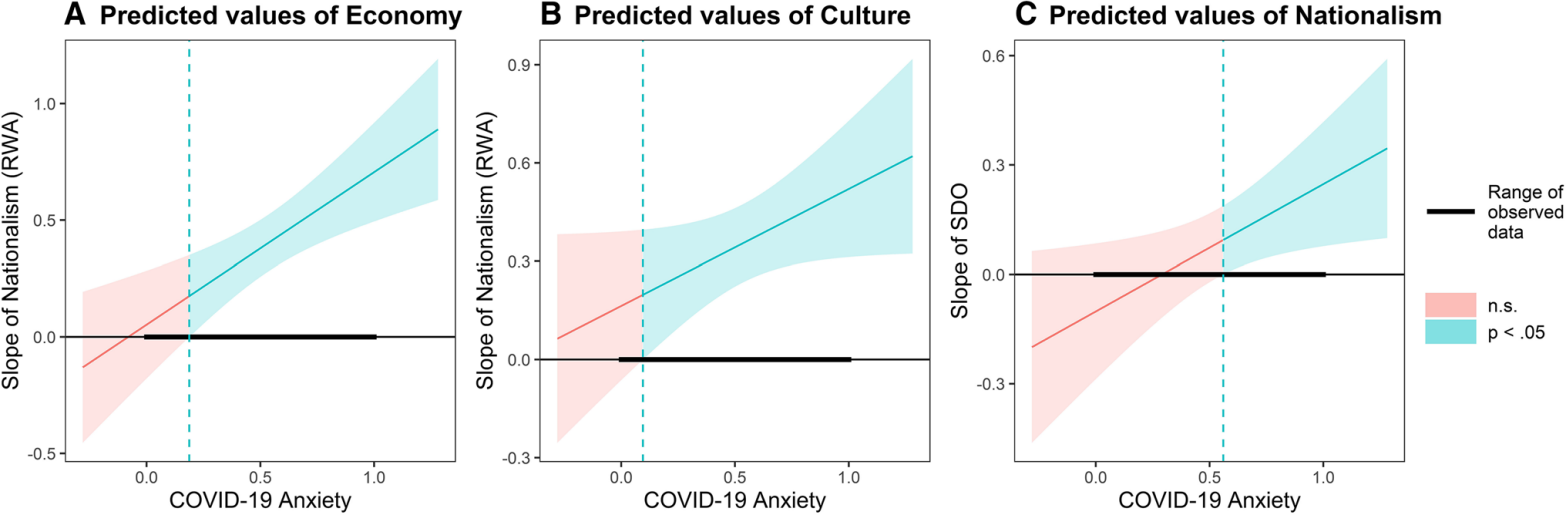
Graph of the Johnson-Neyman region of significance for the interaction effects

The slope of RWA on anti-immigrant sentiments about the economy is statistically significant (with *p* < .05) when COVID-19 anxiety is above 0.19. The slope of RWA on anti-immigrant sentiments about culture is statistically significant (with *p* < .05), when COVID-19 anxiety is above 0.10. The slope of SDO on Nationalism is statistically significant (with *p* < .05), when COVID-19 anxiety is above 0.56. Figure 2 shows the Johnson-Neyman plot for anti-immigrant sentiments about the economy, anti-immigrant sentiments about culture, and nationalism, respectively.

Summarizing these results, they show that in the Italian context COVID-19 anxiety strengthens the association between RWA and anti-immigrant sentiments. The interaction between COVID-19 anxiety and RWA is statistically significant on anti-immigrant sentiment towards the national economy (unadj: B = 0.68, *p* < .002, adj: B = 0.65, *p* < .003), but is not statistically significant on the negative impact of immigrants on Italian culture (unadj: B = 0.50 *p* = .055, adj: B = 0.36, *p* = .25).

In terms of social dominance, when nationalism is set as the dependent variable the estimated effect of SDO, while its main effect is absent, increases in conjunction with COVID-19 perceived anxiety (unadj: B = 0.47 *p* < .01). When the covariates are added into the adjusted regression model, the statistical significance of SDO in interaction with COVID-19 anxiety is no longer achieved (adj: B = 0.35 *p* = .11).

When anti-immigrant sentiments about the internal economy and national culture are set as dependent variables in the adjusted regression models, the interaction of SDO and COVID-19 anxiety was in the inverse direction, namely negative (as anxiety increases, the coefficients for SDO decreases): still, it had a not significant effect on the internal economy (adj: B = 0.40 *p* = .09) nor on culture.Considering the complete models (i.e., ‘adjusted’) with the introduction of sociodemographic and political covariates, higher levels of nationalism were observed in older inhabitants (adj: B = 0.11 *p* = .001) and with higher scores of conspiracy beliefs related to COVID-19 (adj: B = 0.19 *p* < .001). Stronger anti-immigrant sentiments towards the internal economy distinguished people with lower income (adj: B = -0.8 *p* = .02) and with a political ideology relatively on the right (adj: B = 0.04, *p* < .001). A more right-wing political position also characterized people who believe in the negative impact of immigrants on Italian culture (adj: B = 0.05 *p* < .001). Interestingly, no differences were observed between Italian regions in none of the regression models.

## Discussions

In the context of the COVID-19 pandemic, this study explored the relationship between threat perception and political attitudes in Italy. We highlight the role of existential pandemic-related threats in fostering Right-wing political attitudes and ethnocentric attitudes. In line with Hartman et al. (2021), the COVID-19 perceived anxiety appears to moderate the association between Right-Wing ideological predispositions (i.e., RWA and SDO) and ethnocentric political attitudes (i.e., nationalism and anti-immigrant sentiments). In an Italian representative sample, we found that both RWA and SDO had a positive and significant association with nationalism and anti-immigrant sentiments. 

Despite at first glance the bivariate associations of COVID-19 anxiety with RWA, SDO, and anti-immigrant sentiments were low, the regression models by considering more variables together allowed to disentangle the association of COVID-19 anxiety and the aforementioned constructs.

 Despite in the regressions with covariates (i.e., sociodemographic and political orientation) the main effects of RWA and SDO on anti-immigrant sentiments were statistically non-significant, considering RWA and SDO in conjunction with COVID-19 related anxiety allowed some significant associations to emerge.

 Interestingly, considering the economy-related field, there was a statistically significant interaction of RWA and the perceived anxiety of COVID-19 (impersonal existential threat) in predicting higher anti-immigrant sentiments. Thus, as predicted on the basis of preexisting literature, the different levels of COVID-19 perceived anxiety changed the relationship between authoritarianism (RWA) and anti-immigrant sentiments. In other words, the higher participants’ perceived threat levels predicted higher RWA anti-immigrant sentiments concerning the economic aspects. Also, the interval of significance of this moderation was for values of COVID-19 anxiety above 0.19. Contrary to the pattern emerged in the Republic of Ireland, the perceived threat of COVID-19 did not moderate the effect of RWA on nationalism and anti-immigrant sentiments concerning cultural aspects, as well as in the UK study (Hartman et al., 2021). Furthermore, in the Italian sample, the perceived pandemic threat (expressed by COVID-19 anxiety) moderated the relationship between SDO and Right-wing attitudes as well, indeed, high levels of perceived threat were predictive of a close relationship between SDO and nationalism. In particular, the association of SDO and nationalism was significantly moderated by COVID-19 anxiety when the latter showed levels above 0.56. In other words, when the perceived threat reflected by COVID-19 anxiety was moderate-to-high, SDO significantly predicted higher levels of nationalism.

 These findings suggest the role of existential threat in enhancing out-group prejudice in the Italian pandemic context. In particular, the present research showed how the anxiety generated by the impersonal COVID-19 threat can have serious consequences for inter-group dynamics by strengthening the right-wing attitudes that could generate outgroup derogation up to exclusion and violent and aggressive behaviors. For instance, during the COVID-19 pandemic, several episodes of violence toward immigrants occurred in Italy (Devakumar et al., 2020; Esses & Hamilton, 2021). Interestingly, the focus of this study was on a generic impersonal threat, and this suggests that even a threat not exerted by any out-group (e.g., illness, catastrophe, crisis) can influence inter-group attitudes (Hartman et al., 2021).

 In a way, the findings of this study are consistent with those in the literature focused on the existential threat, mortality salience, and *conservative shift* (Burke et al., 2010, 2013; Greenberg et al., 1992; Jost et al., 2017). Uncertain and threatening situational factors (Freeston et al., 2020) might enhance anxiety, authoritarianism, prejudice, and suspicion as well. This hypothesis seems consistent with the significant positive association between conspiracy beliefs related to COVID-19 and nationalism that this study found in the Italian population.

 This study has some limitations that should be considered. The cross-sectional study design did not allow to draw any causal inference. Further studies should, therefore, test the direction of influence among variables through experimental and/or longitudinal designs to understand whether perceived threat moderates the effect of RWA or SDO on ethnocentric political attitudes or, vice versa, personal predispositions (i.e., RWA and SDO) moderate the effect between perceived threat and nationalism and anti-immigrant attitudes. Moreover, the existential pandemic-related threat was measured using a single proxy of COVID-19 perceived anxiety, future studies should use a more precise measure with more items to confirm these results.

 Future research may try to replicate these findings by using a longitudinal design to observe the evolutions of constructs over time (Bennett et al., 2023).

 Regarding the strengths of this study, it was one of the first studies highlighting the RWA and SDO dynamics in the Italian context during the COVID-19 pandemic (Bochicchio et al., 2021; Roccato et al., 2021). Furthermore, much of the literature about the impact of COVID-19 focused on the (intra)individual psychological issues, this study is one of the few highlighting its impact on inter-individual relationships and within the whole society. Given the relevance of such issues and the role some situational-impersonal factors might have in shaping political attitudes and intergroup behaviors), further studies are needed to understand the generality of this phenomenon and to understand the role that contextual factors play in intergroup relations.

 Noteworthy, understanding these complex individual, inter-individual, and social dynamics from a theoretical point of view is the first step to plan and actualize interventions to improve the overall well-being of society and individuals (Bochicchio et al., 2021). Indeed, the findings of this study may be useful to inform psychological interventions to reduce and manage the distressing symptoms (as the anxious ones) triggered by a perceived threat, thus indirectly favoring more inclusive attitudes towards the outgroup. Various approaches can be used to elaborate on and accept the threatening situation (Consoli et al., 2020; Sun et al., 2020). For example, according to the Terror Management Theory (Greenberg et al., 1992; Pyszczynski et al., 2004), the buffering effect of self-esteem may be used to hinder the adverse effects of symptoms (e.g., anxious, traumatic) triggered by the impersonal COVID-19 threat (Rossi et al., 2022a, b). Since this kind of psychosocial phenomenon can have serious relapses for society and its components, interventions should be large-scaled to reach a large part of the population. but it is also important to encourage people to seek professional psychological help (Mannarini et al., 2020; Rossi & Mannarini, 2019; Schnyder et al., 2017). In particular, the categories most at-risk for adverse psychological outcomes – as people with previous psychological difficulties (Bottesi et al., 2018; Rossi et al., 2022a) and/or physical issues (Rossi Ferrario et al., 2021; Panzeri & Ferrario, 2020), elderlies (Panzeri et al., 2021a, b, c), and healthcare workers (Panzeri et al., 2021a, b, c) – should be supported by favoring their access to psycho-social support, both in terms of connection with the social community and through psychological interventions (Panzeri et al., 2022; Ratti et al., 2017).

 Among the methodological strengths of this study, in particular, the stratified sampling allowed to gain a sample representative of the Italian population from north to south. Moreover, the replication of the research design used in other countries allowed us to compare the results within the same framework and with fewer confounding factors (Hartman et al., 2021; McBride et al., 2020a, b).

 In conclusion, overall, the findings of this study results might be useful for researchers, clinicians, and policymakers. Only a real dialogue between the academic field and governance can debunk some authoritarian drifts and hostile climates still present in many contexts.

## Supplementary information

Below is the link to the electronic supplementary material.

ESM 1 (DOCX 71.1 KB)

## Data Availability

Data can be accessed upon reasonable request to the corresponding Author.
