# Supplementary material for: The role of the COVID-19 impersonal threat strengthening the associations of right-wing attitudes, nationalism and anti-immigrant sentiments
Source: Curr Psychol. 2023 Feb 2:1–12. Online ahead of print. doi: 10.1007/s12144-023-04305-w (PMC9892674; doi:10.1007/s12144-023-04305-w)
Supplement: Supplementary file 1 — (DOCX 71.1 KB) [file 12144_2023_4305_MOESM1_ESM.docx]

**Supplementary materials**

**The impersonal threat of COVID-19 strengthens the associations among Right-wing Attitudes and nationalism and anti-immigrant sentiments**

1. **Measures**

***Socio-demographic characteristics:*** Biological sex, Age, Ethnicity, Religious affiliation, Highest level of educational attainment, Gross annual household income, Current economic activity, Residential location (urban or rural), Household composition (number of adults and underaged children), Born in Italy and raised there (i.e., lived in Italy before the age of 16 years).

***Health characteristics:*** Precarious health of self, Precarious health of beloved ones, Pregnancy (self and others) and week of gestation.

***COVID-19:***

*Knowledge of COVID-19 symptoms.* Respondents indicated which of 12 symptoms they believed to be the most commonly reported symptoms of COVID-19. Response format: Categories (yes/unsure/no)

*Transmission of COVID-19*. Respondents indicated which of 8 pathways of transmission of COVID-19 they believed were possible. Response format: Categories (yes/no)

*Attitudes relating to risk of contracting COVID-19*. Respondents rated their agreement with 6 statements about reducing the risk of contracting COVID-19 (e.g., washing hands with soap, using alcohol-based hand-rub; maintaining 1 meter between persons). Response format: a 5-point Likert scale ranging from 1 ‘completely disagree’ to ‘5 completely agree’

*Health Behaviours relating to preventing COVID-19 transmission*. Participants rated the extent to which 17 statements based on the COM-B (Capability, Opportunity, Motivation-Behaviour, version 1) (Michie, Van Stralen, & West, 2011) were true for them during the COVID-19 pandemic. The health-protective behaviours were *maintaining hygienic practices*and *social distancing* to help prevent COVID-19 and reduce the spread of the disease. Response format: a 5-point Likert scale ranging from 1 ‘strongly agree’ to 5 ‘strongly disagree’

*Personal behaviour change to reduce individual risk of contracting COVID-19*.  Respondents reported whether and how they changed behaviour to reduce the risk of being infected by COVID-19. Response format: a 4-point Likert scale from 1 ‘Not at all’ to 4 ‘Avoided completely’.

*Experiences of COVID-19 symptoms, testing and diagnosis*. Respondents reported if they experienced symptoms of COVID-19, whether they had been tested for the virus, the outcome (positive/negative) of that test, if someone close to them (e.g., a family member or friend) had experienced symptoms of COVID-19, if the outcome after being tested for COVID-19 was positive or negative, and if they were currently (or had in the recent past) self-isolated. Response format: Categories (yes/unsure/no)

*Anxiety relating to COVID-19*. Respondents’ rated on a visual analogue scale the specific anxiety about the COVID-19 pandemic. Response format: a visual slider scale ranging from 0 ‘not at all anxious’ on the left to 100 ‘extremely anxious’ on the right.

*Perceived risk of contracting COVID-19.*Respondents estimated the risk of contracting COVID-19 within 1 month, within 3 months, and within 6 months. They also estimated the perceived risk of different vulnerable groups (the elderly, children, pregnant women, and those with underlying chronic health conditions) experienced serious illness and (separately) death following a diagnosis of COVID-19. Response format: Visual sliders (ranging from 0% on the left-hand side to 100% on the right-hand side)

*Knowledge of appropriate action to take following COVID-19 diagnosis.* Respondents reported what members of the public had been advised to do if they suspected that they had been infected by COVID-19. Response format: Categories.

*Attitudes relating to accepting any potential COVID-19 vaccine*. Respondents expressed the acceptability of a new COVID-19 vaccine for themselves, their children, their relatives’ children, and their elderly relatives. Response format: Categories (yes/no/maybe)

*Consumer purchasing during COVID-19 pandemic.* Respondents reported the extent to which they increased purchasing of various items (e.g., sanitary products, dried foods) in the weeks before the survey. Response format: a five-point scale ranging from (1) ‘not at all’ to 5 ‘very considerably’.

*Perceived household income changes* *during the COVID-19 pandemic*. 1) Respondents reported their experiences of (a) a loss of income due to not being able to work and about (b) financial savings due to changes in leisure activities. 2) Respondents reported their worries about how the pandemic affected their household finances. Response format: 1) Categories (yes/no/unsure); 2) Response options ranging from 1 ‘not at all worried’ to 10 ‘extremely worried’.

***Mental health***

Depression was assessed with the *Patient Health Questionnaire-9 (PHQ-9*)  (Kroenke, Spitzer, & Williams, 2001) a nine-item measure asking how often, over the last two weeks, each of the depressive symptoms was present. PHQ-9 scores range from 0 to 27, with scores of ≥5, ≥10, ≥15, representing mild, moderate and severe levels of depression severity (Kroenke et al., 2001). A threshold of ≥10 was used in this study. Response format: a 4 point Likert scale with 0 = 'not at all’, 1=‘several days’, 2=‘more than half the days’, and 3=‘nearly every day’.

*Generalized Anxiety Disorder Scale (GAD-7)* (Spitzer, Kroenke, Williams, & Löwe, 2006) assessed the frequency of seven anxiety symptoms in the past 7 days. A cut-off score of 10 identifies generalised anxiety disorder (Spitzer et al., 2006). Response format: a 4-point Likert scale ranging from 1 (not at all) to 4 (nearly every day).

*Persecution and Deservedness Scale* (PaDS) (Melo, Corcoran, Shryane, & Bentall, 2009) assessed paranoia with five items (statements) from the persecution subscale of the persecution and deservedness scale (PaDS) (Elahi, Algorta, Varese, McIntyre, & Bentall, 2017). Response format: a 5-point scale ranging from 1 = strongly disagree to 5 = strongly agree.

*International Trauma Questionnaire (ITQ)* (Cloitre et al., 2018). The ITQ assessed Post-traumatic stress disorder in relation to the COVID-19 experience in the past month with 6 items across the three symptom clusters of Re-experiencing, Avoidance, and Sense of Threat. The functional impairment caused by these symptoms was measured with three items. Response format: a 5-point Likert scale from 0 (Not at all) to 4 (Extremely) with possible PTSD scores ranging from 0 to 24.

*Patient Health Questionnaire-15 (PHQ-15)* (Kroenke, Spitzer, & Williams, 2002): it assesses the presence and severity of somatization disorder somatic symptoms experienced over the last week. Total score ranges from 0 to 30, scores of ≥5, ≥10, ≥15 represent mild, moderate and severe somatization levels. Response format: 0 (‘not bothered at all’), 1 (‘bothered a little’) or 2 (‘bothered a lot’).

*Treatment seeking behaviour for mental health difficulties*.  respondents reported their history of mental health treatment. Response format: Categories (never received; received in the past; currently receiving).

***Psychological variables***

*Death Anxiety Inventory (DAI)*(Tomás-Sábado, Gómez-Benito, & Limonero, 2005). It assessed the respondents’ attitudes towards death by using 17-item across four death anxiety factors (death acceptance, externally generated death anxiety, death finality, thoughts about death). Response format: a 5-point Likert scale ranging from 1 ‘totally disagree’ to 5 ‘totally agree’.

*Big-Five Inventory (BFI-10)* (Rammstedt & John, 2007). It assessed the five personality traits of openness to experience, conscientiousness, extroversion, agreeableness and neuroticism with two items per construct. Response format: a 5-point Likert type scale from 1 ‘totally disagree’ to 5 ‘totally agree’.

*Intolerance of uncertainty* *Scale* (IUS) (Buhr & Dugas, 2002). It evaluates the intolerance of uncertainty that generates and maintain worry, with 12 items. Response format: a 5-point Likert scale ranging from 1 ‘not at all characteristic of me’ to 5 ‘entirely characteristic of me’.

*Loneliness Scale* (Hughes, Waite, Hawkley, & Cacioppo, 2004). It measures social connectedness with 3 items about the frequency of loneliness. Response format: a 3-point scale (hardly ever, sometimes, or often).

*Obsessive-Compulsive Inventory-Revised*(OCI-R***)***(Marchetti, Chiri, Ghisi, & Sica, 2010)***.***With 18 items it measures the principal 6 dimensions characterizing the obsessive-compulsive disorder:*‘Washing’, ‘Obsessing’, ‘Hoarding’, ‘Ordering’, ‘Checking’, and ‘Mental Neutralizing’.*Response format: a 5-point Likert type scale ranging from 0 (bothered not at all) to 4 (bothered a lot).

*Single-Item Self-esteem Scale (SISES*) (Robins, Hendin, & Trzesniewski, 2001). Respondents’ reported their level of agreement with a single statement (‘*I have high self-esteem’*). Response format: a 7-point Likert scale ranging from 1 ‘not very true of me’ to 7 ‘very true of me’.

*Brief Resilience Scale (BRS)* (B. W. Smith et al., 2008). It assesses the level of trait resilience with 6 items, such as *‘I tend to bounce back quickly after hard times’.*Response format: a 5-point Likert scale ranging from 1 ‘strongly disagree’ to 5 ‘strongly agree’.

*Cognitive Reflection Task of Analytical Reasoning (CRT) (*Frederick, 2005) measures analytical reasoning ability with three problems and two additional ones. Each problem stimulates intuitive but incorrect answers whilst analytic reasoning. Response format: multiple choice with three foil answers (including the hinted incorrect answer).

***Social and political attitudes and behaviours***

*Very Short Authoritarianism Scale (VSA)* (Bizumic & Duckitt, 2018). The VSA was used to assess respondents’ levels of right-wing authoritarianism (RWA) and includes 6 items such as: *‘It’s great that many young people today are prepared to defy authority*’; ‘*What our country needs most is discipline, with everyone following our leaders in unity*’; and ‘*Our society does NOT need tougher government and stricter laws’*. Response format: a 5-point Likert scale ranging from 1 ‘strongly disagree’ to 5 ‘strongly agree’.

*Social Dominance Scale* (SDO_7_) (Ho et al., 2015).  Social dominance orientation levels were assessed using the SDO7 with 8 items such as *‘Some groups of people are simply inferior to other groups’.*Response format: a 7-point Likert scale ranging from 1 ‘Strongly oppose’ to 5 ‘Strongly Favour’.

*Identification with all humanity scale (IWAH)* (McFarland, Webb, & Brown, 2012). The nine items were adapted for use in this study with reference to the ‘Italians’. Respondents reported how much they felt identified with people in their community; people from Italy; and all humans everywhere. Response format: from 1 ‘not at all’ to 5 ‘very much’.

*Patriotism/Nationalism*. Patriotism (three items) was referred to as ‘constructive nationalism’ has been conceptualised as love for one’s country, whereas nationalism (two items) has been conceptualised as the belief that one’s country is superior to others. Items to measure patriotism and nationalism were adapted from Davidov (2011). Response format: a 5-point Likert scales from 1 ’strongly disagree’ to 5 ’strongly agree’.

*Attitude towards migrants*. It assessed attitudes towards migrants with 3 items adapted from the British Social Attitudes Survey 2015 (British Social Attitudes Survey 2015, 2015): (1) ‘*Would you say it is generally bad or good for Italy’s economy that migrants come to Italy from other countries*?’ (2) ‘*Would you say that Italy’s cultural life is generally undermined or enriched by migrants coming to live here from other countries?*’ and (3) ‘*Some migrants make use of Italy’s schools, increasing the demand on them. However, many migrants also pay taxes which support schools and some also work in schools. Do you think that, on balance, migration to Italy reduces or increases pressure on the schools*?’. Response format: 1) scored on a 10-point scale ranging from 1 ‘extremely bad’ to 10 ‘extremely good’; 2) scored on a 10-point scale ranging from 1 ‘undermined’ to 10 ‘enriched’; 3) scored on a 5-point scale ranging from 1 ‘reduces pressure a lot’ to 5 ‘increases pressure a lot’?

*Voting behaviour and political party affiliation.* Respondents reported: (1) if they had voted the last time; 2) which political party they voted for; (3) their political affiliation, (4) their views on social issues such as abortion and same-sex marriage and (5) their views on economic issues such as taxes and government spending. Response format: (1) categorial (yes/no); (2) categorial (political parties); (3) a 10-point scale ranging from 1 ‘left-wing’ to 10 ‘right-wing’; (4) and (5) a 10-point scale from 1 ‘very liberal’ to 10 ‘very conservative’.

*Conspiracy mentality scale (CMS)* (Imhoff & Bruder, 2014). It evaluates a generalized political attitude with five items such as: *‘I think that there are secret organizations that greatly influence political decisions’*. Response format: a 5-point scale from 1 ‘Certainly not 0%’ to 11 ‘Certainly 100%’

*Trust in institutions*. Respondents rated the extent to which they have trust in the institutions/groups such as political parties, government, scientists and health professionals. Response format: a 5-point Likert scale ranging from 1 ‘completely trust’ to 5 ‘do not trust at all’.

*Belongingness in neighbourhood*. It assessed the level of belongingness and connectedness to the neighbourhood with three questions taken from the UK Community Liver Survey (Cabinet Office, 2015): *(1) ‘How strongly do you feel you belong to your immediate neighbourhood?’; (2) ‘How comfortable would you be with asking a neighbour to keep a set of keys to your home for emergencies?’; (3) ‘How comfortable would you be asking a neighbour to collect a few shopping essentials for you, if you were ill and at home on your own?’.*Response format: 1) a 4-point scale from 1 ‘not at all’ to 4 ‘very strongly’; 2) a 4-point scale ranging from 1 ‘very uncomfortable’ to 4 ‘very comfortable’; 3) a 4-point scale ranging from 1 ‘very uncomfortable’ to 4 ‘very comfortable’.

*Religious identity and belief*. Participants selected their religious identity among ‘atheist’, ‘agnostic’ and ‘other’. Also, the Monotheist and Atheist Beliefs Scale (Alsuhibani, Shevlin, & Bentall, 2020, as cited in McBride et al., 2020) has 8 statements measuring religiosity (4 items) vs atheism (4 items). Response format: 5-point Likert scale, from 1 (strongly agree) to 5 (strongly disagree).

1. **Tables**

**Table S1: Age**

|  | N |  | min | 1st Quartile | Mean | Median | 3rd Quartile | max | Std Deviation |
| --- | --- | --- | --- | --- | --- | --- | --- | --- | --- |
|  |  |  |  |  |  |  |  |  |  |
| Overall | 1038 |  | 18 | 34 | 48.2 | 49 | 62 | 87 | 16.14 |
|  |  |  |  |  |  |  |  |  |  |
| Gender |  |  |  |  |  |  |  |  |  |
| F | 531 |  | 18 | 34 | 47.02 | 47 | 60 | 81 | 15.36 |
| M | 507 |  | 18 | 41 | 53 | 55 | 67 | 87 | 16.39 |
|  |  |  |  |  |  |  |  |  |  |
| Region |  |  |  |  |  |  |  |  |  |
| Campania | 227 |  | 18 | 34 | 48.2 | 49 | 62 | 78 | 16.01 |
| Lazio | 234 |  | 20 | 40.25 | 51.77 | 54 | 64 | 87 | 15.46 |
| Lombardia | 391 |  | 18 | 36 | 50.76 | 51 | 66 | 86 | 16.33 |
| Veneto | 186 |  | 20 | 34 | 48.04 | 50 | 61 | 83 | 16.47 |

**Table S2:**Level of Education (frequencies)

|  | N |  | None | Elementary | Secondary | High  School | Bachelor | Master  of Science | PhD | Professional  Qualification |
| --- | --- | --- | --- | --- | --- | --- | --- | --- | --- | --- |
|  |  |  |  |  |  |  |  |  |  |  |
| Overall | 1038 |  | 0 | 3 | 83 | 506 | 97 | 297 | 28 | 24 |
|  |  |  |  |  |  |  |  |  |  |  |
| Gender |  |  |  |  |  |  |  |  |  |  |
| F | 531 |  | 0 | 0 | 43 | 261 | 48 | 151 | 18 | 10 |
| M | 507 |  | 0 | 3 | 40 | 245 | 49 | 146 | 10 | 14 |
|  |  |  |  |  |  |  |  |  |  |  |
| Region |  |  |  |  |  |  |  |  |  |  |
| Campania | 227 |  | 0 | 0 | 13 | 127 | 17 | 62 | 5 | 3 |
| Lazio | 234 |  | 0 | 1 | 16 | 111 | 17 | 77 | 7 | 5 |
| Lombardia | 391 |  | 0 | 1 | 35 | 176 | 39 | 114 | 11 | 15 |
| Veneto | 186 |  | 0 | 1 | 19 | 92 | 24 | 44 | 5 | 1 |

**Table S3:**Income (frequencies)

|  | N |  | –  15.000 €/year | -  28.000  €/year | -  55.000  €/year | -  75.000  €/year | +  75.000  €/year |
| --- | --- | --- | --- | --- | --- | --- | --- |
|  |  |  |  |  |  |  |  |
| Overall | 1038 |  | 218 | 214 | 212 | 211 | 183 |
|  |  |  |  |  |  |  |  |
| Gender |  |  |  |  |  |  |  |
| F | 531 |  | 131 | 118 | 104 | 100 | 78 |
| M | 507 |  | 87 | 96 | 108 | 111 | 105 |
| Region |  |  |  |  |  |  |  |
| Campania | 227 |  | 72 | 59 | 39 | 23 | 34 |
| Lazio | 234 |  | 56 | 36 | 44 | 57 | 41 |
| Lombardia | 391 |  | 55 | 74 | 91 | 91 | 80 |
| Veneto | 186 |  | 35 | 45 | 38 | 40 | 28 |

**Table S4:**Covid-19 total participants tested & confirmed cases (participants, relatives, friends)

|  | N |  | Personal,  Total Tested | Cases | | |
| --- | --- | --- | --- | --- | --- | --- |
|  |  |  |  | **Personal, confirmed** | **Relatives, confirmed** | **Friends,**  **confirmed** |
|  |  |  |  |  |  |  |
| Overall | 1038 |  | 141 | 14 | 26 | 185 |
|  |  |  |  |  |  |  |
| Gender |  |  |  |  |  |  |
| F | 531 |  | 74 | 7 | 10 | 98 |
| M | 507 |  | 67 | 7 | 16 | 87 |
|  |  |  |  |  |  |  |
| Region |  |  |  |  |  |  |
| Campania | 227 |  | 22 | 1 | 6 | 31 |
| Lazio | 234 |  | 29 | 2 | 3 | 28 |
| Lombardia | 391 |  | 66 | 7 | 14 | 97 |
| Veneto | 186 |  | 24 | 4 | 3 | 29 |

**Table S5:**Right-wing authoritarianism (6-30)

|  | N |  | min | 1st Quartile | Mean | Median | 3rd Quartile | max | Std Deviation |
| --- | --- | --- | --- | --- | --- | --- | --- | --- | --- |
|  |  |  |  |  |  |  |  |  |  |
| Overall | 1038 |  | 6 | 16 | 17.9 | 18 | 20 | 30 | 3.84 |
|  |  |  |  |  |  |  |  |  |  |
| Gender |  |  |  |  |  |  |  |  |  |
| F | 531 |  | 8 | 16 | 17.77 | 18 | 20 | 28 | 3.71 |
| M | 507 |  | 6 | 16 | 18.03 | 18 | 20 | 30 | 3.97 |
|  |  |  |  |  |  |  |  |  |  |
| Region |  |  |  |  |  |  |  |  |  |
| Campania | 227 |  | 6 | 16 | 17.78 | 18 | 20 | 28 | 3.82 |
| Lazio | 234 |  | 7 | 16 | 17.93 | 18 | 20 | 30 | 4.10 |
| Lombardia | 391 |  | 7 | 15.50 | 17.75 | 18 | 20 | 29 | 3.71 |
| Veneto | 186 |  | 8 | 16 | 18.31 | 18 | 20 | 28 | 3.79 |

**Table S6:**Social dominance orientation (8-56)

|  | N |  | min | 1st Quartile | Mean | Median | 3rd Quartile | max | Std Deviation |
| --- | --- | --- | --- | --- | --- | --- | --- | --- | --- |
|  |  |  |  |  |  |  |  |  |  |
| Overall | 1038 |  | 8 | 19 | 24.92 | 26 | 31 | 56 | 7.43 |
|  |  |  |  |  |  |  |  |  |  |
| Gender |  |  |  |  |  |  |  |  |  |
| F | 531 |  | 8 | 19 | 25.02 | 26 | 32 | 42 | 7.45 |
| M | 507 |  | 8 | 19 | 24.82 | 25 | 31 | 56 | 7.43 |
|  |  |  |  |  |  |  |  |  |  |
| Region |  |  |  |  |  |  |  |  |  |
| Campania | 227 |  | 8 | 17 | 23.34 | 24 | 31 | 37 | 7.79 |
| Lazio | 234 |  | 8 | 18 | 24.41 | 25 | 31 | 56 | 7.87 |
| Lombardia | 391 |  | 8 | 20 | 25.28 | 26 | 32 | 45 | 7.10 |
| Veneto | 186 |  | 8 | 23 | 26.76 | 28 | 32 | 42 | 6.66 |

**Table S7:**Covid-19 Related Anxiety (0-100)

|  | N |  | min | 1st Quartile | Mean | Median | 3rd Quartile | max | Std Deviation |
| --- | --- | --- | --- | --- | --- | --- | --- | --- | --- |
|  |  |  |  |  |  |  |  |  |  |
| Overall | 1038 |  | 0 | 30 | 54.21 | 60 | 76 | 100 | 28.04 |
|  |  |  |  |  |  |  |  |  |  |
| Gender |  |  |  |  |  |  |  |  |  |
| F | 531 |  | 0 | 44 | 59.99 | 63 | 80 | 100 | 26.39 |
| M | 507 |  | 0 | 20 | 48.15 | 51 | 71 | 100 | 28.47 |
|  |  |  |  |  |  |  |  |  |  |
| Region |  |  |  |  |  |  |  |  |  |
| Campania | 227 |  | 0 | 42 | 57.95 | 61 | 79.50 | 100 | 27.09 |
| Lazio | 234 |  | 0 | 30 | 53.96 | 59.50 | 75 | 100 | 28.88 |
| Lombardia | 391 |  | 0 | 30 | 52.79 | 60 | 76 | 100 | 28.00 |
| Veneto | 186 |  | 0 | 30 | 52.93 | 51 | 75 | 100 | 28.01 |

**Table S8:**Conspiracy (rescaled, 0-100)

|  | N |  | min | 1st Quartile | Mean | Median | 3rd Quartile | max | Std Deviation |
| --- | --- | --- | --- | --- | --- | --- | --- | --- | --- |
|  |  |  |  |  |  |  |  |  |  |
| Overall | 1038 |  | 0 | 18.60 | 32.02 | 28 | 43 | 100 | 19.77 |
|  |  |  |  |  |  |  |  |  |  |
| Gender |  |  |  |  |  |  |  |  |  |
| F | 531 |  | 0 | 19.50 | 33.18 | 29 | 45 | 100 | 20.75 |
| M | 507 |  | 0 | 18.20 | 30.80 | 27.10 | 40.15 | 100 | 21.47 |
|  |  |  |  |  |  |  |  |  |  |
| Region |  |  |  |  |  |  |  |  |  |
| Campania | 227 |  | 0 | 18.70 | 33.15 | 29 | 44 | 100 | 20.24 |
| Lazio | 234 |  | 0 | 17.60 | 30.60 | 26.50 | 41.95 | 100 | 19.10 |
| Lombardia | 391 |  | 0 | 18.60 | 31.50 | 28.10 | 42 | 100 | 20.03 |
| Veneto | 186 |  | 0 | 20.00 | 33.50 | 29.20 | 45.15 | 100 | 20.80 |

**Table S9:**Political view (left to right, 1-10)

|  | N |  | min | 1st Quartile | Mean | Median | 3rd Quartile | max | Std Deviation |
| --- | --- | --- | --- | --- | --- | --- | --- | --- | --- |
|  |  |  |  |  |  |  |  |  |  |
| Overall | 1038 |  | 1 | 3 | 5.47 | 5 | 8 | 10 | 2.60 |
|  |  |  |  |  |  |  |  |  |  |
| Gender |  |  |  |  |  |  |  |  |  |
| F | 531 |  | 1 | 3 | 5.47 | 6 | 8 | 10 | 2.65 |
| M | 507 |  | 1 | 3 | 5.47 | 5 | 8 | 10 | 2.55 |
|  |  |  |  |  |  |  |  |  |  |
| Region |  |  |  |  |  |  |  |  |  |
| Campania | 227 |  | 1 | 3 | 5.13 | 5 | 7 | 10 | 2.50 |
| Lazio | 234 |  | 1 | 3 | 5.25 | 5 | 8 | 10 | 2.74 |
| Lombardia | 391 |  | 1 | 3.5 | 5,48 | 5 | 8 | 10 | 2.56 |
| Veneto | 186 |  | 1 | 5 | 6.13 | 6 | 8 | 10 | 2.51 |

**Table S10:**Ideological orientation toward social issues (left to right, 1-10)

|  | N |  | min | 1st Quartile | Mean | Median | 3rd Quartile | max | Std Deviation |
| --- | --- | --- | --- | --- | --- | --- | --- | --- | --- |
| Overall | 1038 |  | 1 | 1 | 4.19 | 4 | 6 | 10 | 2.82 |
|  |  |  |  |  |  |  |  |  |  |
| Gender |  |  |  |  |  |  |  |  |  |
| F | 531 |  | 1 | 1 | 3.96 | 4 | 6 | 10 | 2.72 |
| M | 507 |  | 1 | 2 | 4.43 | 4 | 7 | 10 | 2.90 |
|  |  |  |  |  |  |  |  |  |  |
| Region |  |  |  |  |  |  |  |  |  |
| Campania | 227 |  | 1 | 1 | 4.15 | 4 | 6 | 10 | 2.75 |
| Lazio | 234 |  | 1 | 1 | 4.18 | 4 | 7 | 10 | 3.00 |
| Lombardia | 391 |  | 1 | 1 | 4.04 | 4 | 6 | 10 | 2.68 |
| Veneto | 186 |  | 1 | 2 | 4.57 | 4 | 7 | 10 | 2.93 |

**Table S11:**Ideological orientation toward fiscal issues (left to right, 1-10)

|  | N |  | min | 1st Quartile | Mean | Median | 3rd Quartile | max | Std Deviation |
| --- | --- | --- | --- | --- | --- | --- | --- | --- | --- |
| Overall | 1038 |  | 1 | 3 | 4.62 | 5 | 6 | 10 | 2.33 |
|  |  |  |  |  |  |  |  |  |  |
| Gender |  |  |  |  |  |  |  |  |  |
| F | 531 |  | 1 | 3 | 4.60 | 5 | 6 | 10 | 2.26 |
| M | 507 |  | 1 | 3 | 4.63 | 5 | 6 | 10 | 2.41 |
|  |  |  |  |  |  |  |  |  |  |
| Region |  |  |  |  |  |  |  |  |  |
| Campania | 227 |  | 1 | 3 | 4.35 | 4 | 6 | 10 | 2.25 |
| Lazio | 234 |  | 1 | 3 | 4.85 | 5 | 7 | 10 | 2.62 |
| Lombardia | 391 |  | 1 | 3 | 4.37 | 4 | 6 | 10 | 2.19 |
| Veneto | 186 |  | 1 | 4 | 5.16 | 5 | 6 | 10 | 2.25 |

**Table S12:**Nationalism (low to high, 2-10)

|  | N |  | min | 1st Quartile | Mean | Median | 3rd Quartile | max | Std Deviation |
| --- | --- | --- | --- | --- | --- | --- | --- | --- | --- |
| Overall | 1038 |  | 2 | 5 | 6.13 | 6 | 7 | 10 | 1.79 |
|  |  |  |  |  |  |  |  |  |  |
| Gender |  |  |  |  |  |  |  |  |  |
| F | 531 |  | 2 | 5 | 6.03 | 6 | 7 | 10 | 1.79 |
| M | 507 |  | 2 | 5 | 6.23 | 6 | 7 | 10 | 1.79 |
|  |  |  |  |  |  |  |  |  |  |
| Region |  |  |  |  |  |  |  |  |  |
| Campania | 227 |  | 2 | 5 | 5.05 | 6 | 7 | 10 | 1.86 |
| Lazio | 234 |  | 2 | 5 | 6.17 | 6 | 7 | 10 | 1.76 |
| Lombardia | 391 |  | 2 | 5 | 6.13 | 6 | 7 | 10 | 1.78 |
| Veneto | 186 |  | 2 | 5 | 6.16 | 6 | 7 | 10 | 1.77 |

**Table S13:**Anti-immigrant sentiment: Economy (low to high, 1-10)

|  | N |  | min | 1st Quartile | Mean | Median | 3rd Quartile | max | Std Deviation |
| --- | --- | --- | --- | --- | --- | --- | --- | --- | --- |
|  |  |  |  |  |  |  |  |  |  |
| Overall | 1038 |  | 1 | 3 | 5.14 | 5 | 7 | 10 | 2.78 |
|  |  |  |  |  |  |  |  |  |  |
| Gender |  |  |  |  |  |  |  |  |  |
| F | 531 |  | 1 | 2.5 | 5 | 5 | 7 | 10 | 2.79 |
| M | 507 |  | 1 | 3 | 5.29 | 5 | 8 | 10 | 2.76 |
|  |  |  |  |  |  |  |  |  |  |
| Region |  |  |  |  |  |  |  |  |  |
| Campania | 227 |  | 1 | 3 | 5.42 | 6 | 7 | 10 | 2.65 |
| Lazio | 234 |  | 1 | 2.25 | 5.08 | 5 | 7 | 10 | 2.88 |
| Lombardia | 391 |  | 1 | 3 | 5.11 | 5 | 7 | 10 | 2.78 |
| Veneto | 186 |  | 1 | 2.25 | 4.94 | 5 | 7 | 10 | 2.79 |

**Table S14:**Anti-immigrant sentiment: Culture (low to high, 1-10)

|  | N |  | min | 1st Quartile | Mean | Median | 3rd Quartile | max | Std Deviation |
| --- | --- | --- | --- | --- | --- | --- | --- | --- | --- |
| Overall | 1038 |  | 1 | 3 | 5.39 | 6 | 8 | 10 | 2.84 |
|  |  |  |  |  |  |  |  |  |  |
| Gender |  |  |  |  |  |  |  |  |  |
| F | 531 |  | 1 | 3 | 5.55 | 6 | 8 | 10 | 2.87 |
| M | 507 |  | 1 | 3 | 5.23 | 5 | 7 | 10 | 2.80 |
|  |  |  |  |  |  |  |  |  |  |
| Region |  |  |  |  |  |  |  |  |  |
| Campania | 227 |  | 1 | 4 | 5.74 | 6 | 8 | 10 | 2.77 |
| Lazio | 234 |  | 1 | 3 | 5.40 | 6 | 8 | 10 | 2.86 |
| Lombardia | 391 |  | 1 | 3 | 5.22 | 5 | 7 | 10 | 2.83 |
| Veneto | 186 |  | 1 | 3 | 5.32 | 5 | 8 | 10 | 2.89 |
